# Dietary Choline Protects Against Cognitive Decline After Surgery in Mice

**DOI:** 10.3389/fncel.2021.671506

**Published:** 2021-12-14

**Authors:** Sara V. Maurer, Cuicui Kong, Niccolò Terrando, Christina L. Williams

**Affiliations:** ^1^Department of Psychology and Neuroscience, Duke University, Durham, NC, United States; ^2^Department of Psychiatry, Carver College of Medicine, University of Iowa, Iowa City, IA, United States; ^3^Department of Anesthesiology, Center for Translational Pain Medicine, Duke University School of Medicine, Durham, NC, United States

**Keywords:** choline, PNDs, neuroinflammation, diet, memory, hilus

## Abstract

Perioperative neurocognitive disorders (PNDs) are a common complication following procedures such as orthopedic surgery. Using a mouse model of tibial fracture and repair surgery, we have previously shown an increase in neuroinflammation and hippocampal-dependent cognitive deficits. These changes were ameliorated with the addition of a cholinergic agonist. Here, we sought to examine the effects of a high-choline diet for 3 weeks prior to tibial fracture surgery. We evaluated memory using novel object recognition (NOR) as well as young neurons and glial cell morphology at 1 day and 2 weeks post-surgery. At both time points, tibial fracture impaired NOR performance, and dietary choline rescued these impairments. Astrocytic density and hilar granule cells increased 1 day after tibial fracture, and these increases were partially blunted by dietary choline. An increase in young neurons in the subgranular zone of the dentate gyrus was found 2 weeks after tibial fracture. This increase was partially blunted by choline supplementation. This suggests that shortly after tibial fracture, hippocampal reorganization is a possible mechanism for acute impaired memory. These findings together suggest that non-pharmaceutical approaches, such as pre-surgical dietary intervention with choline, may be able to prevent PNDs.

## Introduction

Perioperative neurocognitive disorders (PNDs) are common following major surgery, hospitalization, and critical illness (Eckenhoff et al., 2020). Patients of all ages are affected by PNDs, but older adults are particularly susceptible and experience worse outcomes (Monk et al., 2008). PNDs are a pervasive health problem with unknown mechanisms and limited understanding of long-lasting effects from anesthesia and surgery.

Neuroinflammation has been suggested as a key driver of acute PNDs (Yang et al., 2020). Using a mouse model of orthopedic surgery, we have shown age-dependent changes in microglial morphology (Xiong et al., 2018) and acute neuroinflammation in the hippocampus (Velagapudi et al., 2019). Blocking this postoperative neuroinflammatory response using selective cholinergic agonists provided anti-inflammatory effects and rescued contextual memory impairments 3 days after tibial fracture (Terrando et al., 2011). Agonists may be exerting this anti-inflammatory effect *via* cholinergic receptors on hippocampal microglia (Abbas et al., 2019), astrocytes (Sharma and Vijayaraghavan, 2001; Papouin et al., 2017), and neurons (Fabian-Fine et al., 2001; Liu et al., 2009).

Research using other surgical models of PNDs have detected cognitive impairments as late as 1 week after surgery in mice (Liang et al., 2018), and 2 weeks in rats (Hovens et al., 2015). However, the longer-lasting effects of surgery remain poorly studied. We hypothesize that, although neuroinflammation may subside within a few days after surgery, neuronal changes may outlast neuroinflammation – thus contributing to longer-lasting cognitive impairments. We sought to quantify behavioral, neuronal, and neuroimmune cell alterations after tibial fracture surgery and implement a dietary intervention using choline prior to surgery.

Choline has well-established anti-inflammatory effects, and dietary choline has been previously shown to be beneficial for hippocampal-dependent memory (Wong-Goodrich et al., 2008). Indeed, dietary choline supplementation increases hippocampal neurogenesis and improves spatial memory in adult rats (Wong-Goodrich et al., 2008). Dietary choline supplementation has also been shown to be protective against dementia in humans (Ylilauri et al., 2019), and spatial memory loss and amyloid-β plaque load in a mouse model of Alzheimer’s disease (Velazquez et al., 2019). In addition, *in vitro* choline supplementation leads to normalization of APOE4-expressing astrocytes (Sienski et al., 2021). As well, in the absence of a neural assault, dietary choline increases memory retention capabilities in adult rats (Moreno et al., 2018). Increased nutritional choline intake and higher free plasma choline levels have also been associated with enhanced episodic memory, verbal memory, and visual memory in human adults (Poly et al., 2011; Nurk et al., 2013). Previous work in our lab and others have found that prenatal and postnatal dietary choline supplementation increases adult neurogenesis (Glenn et al., 2007; Kim et al., 2019). For this reason, we focused on the dentate gyrus of the hippocampus to assess neuroinflammation and cell proliferation within the neurogenic area. Finally, serum choline levels are dramatically decreased after surgery, suggesting that dietary choline supplementation may be making up for this loss (Ulus et al., 1998).

Overall, these data led us to hypothesize that treatment with dietary choline prior to tibial fracture may curtail PND development by blunting changes in neuroinflammation and reactive neurogenesis due to tibial fracture.

## Materials and Methods

**FIGURE 1:**
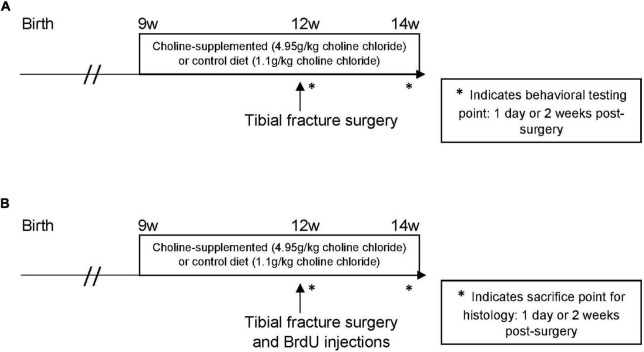
Experimental timelines. **(A)** The effect of choline supplementation and tibial fracture surgery on hippocampal-dependent behavior. Nine-week-old C57/BL6J male mice consumed a synthetic diet that was supplemented with choline or a synthetic control diet without added choline. These diets were consumed for 3 weeks before a tibial fracture surgery. All mice underwent behavioral testing (NOR and open field) 1 day after surgery, with a habituation trial the day of surgery. Mice received another 2 weeks of choline-supplemented or control diet after surgery. After these 2 weeks, they underwent the same behavioral testing. * indicates behavioral testing point: 1 day or 2 weeks post-surgery. **(B)** The effect of choline supplementation and tibial fracture on histological measures. Nine-week-old C57/BL6J male mice were treated with a choline-supplemented diet or a synthetic control diet for 3 weeks before a tibial fracture surgery. The first group of mice was euthanized 1 day after surgery to analyze microglia, astrocytes, and young neurons. The second group received another 2 weeks of choline-supplemented or control diet after surgery. After these 2 weeks, they were euthanized to measure microglia, astrocytes, and young neurons. * indicates sacrifice point for histology: 1 day or 2 weeks post-surgery.

**FIGURE 2:**
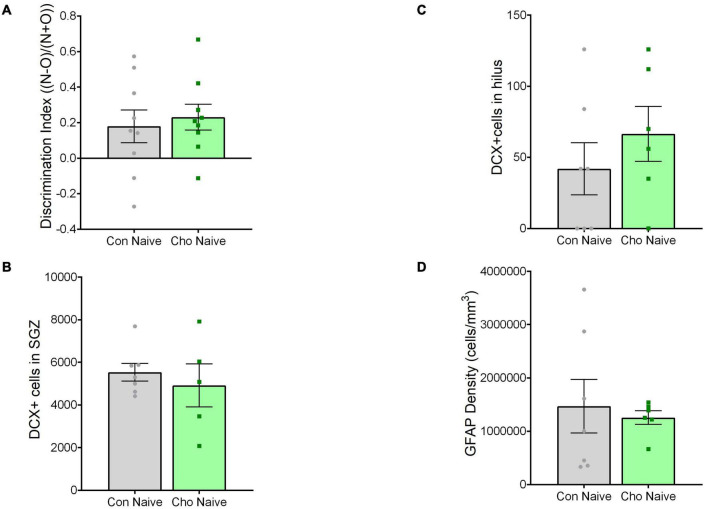
Choline diet alone did not alter hippocampal memory, DCX+ cells, or GFAP antigen density 1 day after surgery. **(A)** Histogram showing discrimination index [(Novel object time − Old object time)/(Novel object time + Old object time)] 1 day after surgery. *N* = 9 per group. **(B)** Histogram showing the number of DCX+ cells in the subgranular zone (SGZ) of the dentate gyrus. *N* = 5–7 per group. **(C)** Histogram showing the number of DCX+ cells in the hilus of the dentate gyrus. *N* = 6–7 per group. **(D)** Histogram showing the antigen density of GFAP in the dentate gyrus of the hippocampus. *N* = 7 per group. Each bar represents the mean ± SEM. Con Naïve, control diet + naïve; Cho Naïve, choline diet + naïve.

Experiments were conducted under the approval of the Duke University Institutional Animal Care and Use Committee. This study was performed using two cohorts of mice, both treated identically: the first group underwent behavioral training, and the second was used for histological analysis (Figure 1). For both cohorts, the experimental design was 3 (treatment condition: control diet, control diet + tibial fracture, choline supplemented diet + tibial fracture) × 2 (time point: 1 day after tibial fracture, 2 weeks after tibial fracture). Because our initial findings showed no differences between “control diet + naïve” and “choline diet + naïve” outcomes 1 day after surgery (Figure 2), the “choline diet + naïve” group was not analyzed further.

### Mice

Nine-week old male C57BL/6J mice (sires were purchased from Jackson Laboratory, Bar Harbor, ME, United States) were bred, raised, and housed in the Duke University vivarium. Mice were housed in groups of 2–5 and kept in individually ventilated, polypropylene shoebox cages with 6.35 mm corn-cob bedding in a room with a 12-h dark-light cycle with lights off at 12 p.m. Filtered water and food was available *ad libitum*.

### Choline Supplementation

Beginning at 9 weeks of age, mice were given their assigned diet until sacrifice at either 1 day or 2 weeks post-tibial fracture surgery. Mice in both experimental cohorts were assigned to one of two *ad libitum* diet conditions: a synthetic control chow (1.1 g/kg choline chloride in formula AIN-76A with choline chloride substituted for choline bitartrate, DYET #110098, Dyets, Inc., Bethlehem, PA, United States), or the same diet with 4.95 g/kg choline chloride (DYET #110210). These diets were chosen based on findings from previous work using these diets (Meck and Williams, 1999; Glenn et al., 2007, 2008, 2012; Wong-Goodrich et al., 2008; Velazquez et al., 2013; Yan et al., 2014; Nickerson et al., 2017). All mice were raised eating standard rodent chow (approximately 2.2 g/kg choline chloride; PicoLab Mouse Diet 5058, Lab-Diet, Philadelphia, PA, United States) before 9 weeks of age.

### Tibial Fracture Surgery

At 12 weeks of age, mice were assigned to either a naïve group (no anesthesia or surgery) or a tibial fracture group. Although sham surgery (anesthesia + skin incision) can trigger mild changes in inflammation and neuronal injury markers (Zhang et al., 2016), we previously found little to no effects of brief (∼10 min) exposure to isoflurane on neuroinflammatory and behavioral endpoints (Cibelli et al., 2010). Thus, we decided to use naïve mice as the control group. Mice in the tibial fracture group were anesthetized with 2.1% isoflurane (Isothesia; Butler Animal Health Supply, Dublin, OH, United States) in 0.30 FiO_2_ prior to receiving a tibial fracture of the left hind leg with an intramedullary fixation as previously described (Harry et al., 2008; Cibelli et al., 2010). Briefly, the left hind leg was disinfected with povidone iodine (Dynarex, Orangeburg, NY, United States) while on a heating pad. Mice were given buprenorphine-SR (0.1 mg/kg, ZooPharm, Laramie, WY, United States) for analgesia. A median incision on the left hind leg was performed, then a 0.38 mm pin was inserted in the intramedullary canal. After insertion, the periosteum was stripped, and osteotomy was performed. Post-fracture, bupivacaine (0.25%; McKesson, Irving, TX, United States) was administered in the wound, and the skin was sutured. Mice recovered from anesthesia in a clean cage before being returned to their home cage and were carefully monitored until it was clear they were eating and drinking normally.

### Behavioral Testing

**FIGURE 3:**
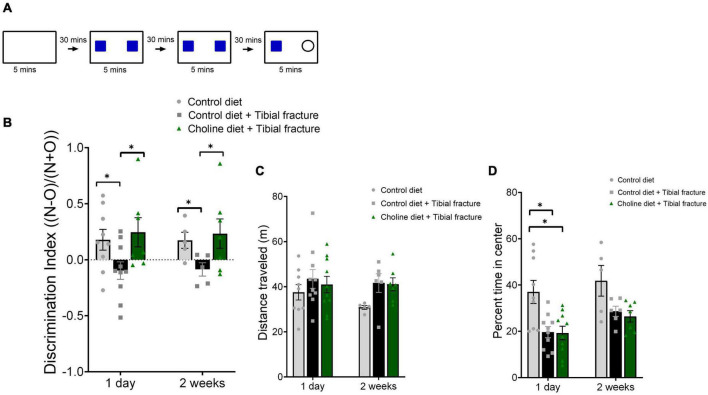
**(A)** Schematic indicating novel object recognition (NOR) protocol. **(B)** Histogram showing discrimination index [(Novel object time − Old object time)/(Novel object time + Old object time)] 1 day and 2 weeks after surgery. *N* = 5–10 per group. *p < 0.01, Tukey HSD. Each bar represents the mean ± SEM. **(C)** Tibial fracture surgery and choline supplemented diet did not alter activity of mice in an open field task. Histogram showing the distance traveled in meters over the 5 min trial. *N* = 5–10 per group. Each bar represents the mean ± SEM. **(D)** Tibial fracture surgery and choline supplemented diet effects on percent of time spent in the center of the arena (anxiety-related behavior) in an open field. Tibial fracture decreased the percent time in the center of the arena, and this effect was not rescued by dietary choline supplementation. Histogram showing the percent of time spent in the center of the arena over the 5 min trial. *N* = 5–10 per group. *p < 0.05, Tukey HSD. Each bar represents the mean ± SEM.

Short term memory for objects was determined using the novel object recognition (NOR) paradigm (modified from Leger et al., 2013; Figure 3A). This task is dependent on the hippocampus when the inter-trial interval (retention interval) is more than 10 mins (reviewed in Cohen and Stackman, 2015). This task was chosen because it does not elicit a stress response and can be refined to assess subtle differences in hippocampal-dependent pattern recognition ability (Tognoni, 2014). Training and testing occurred over 2 days. On the first day each mouse was taken from the colony room, removed from its home cage, and placed in an individual cage in a dark, quiet anteroom for a half hour. Each cage had adequate corncob bedding, the assigned diet, and water in bottles. Each mouse was then brought into the test room and was allowed to habituate for 5 mins in an empty plexiglass arena with opaque walls. The testing room was kept in dim light, in which the ceiling light bulbs were distributed to ensure even light distribution. Light bulbs were a warm white light with 3,000 kelvin color temperature. A plastic tube was used to transport mice from room to room to minimize stress (Hurst and West, 2010). Mice then underwent four trials, each consisting of 5 mins of automated motion tracking (HVS Image, Buckingham, United Kingdom) and video recording in a 406.4 mm L × 203.2 mm W × 254 mm H plexiglass arena on a white table 762 mm above the ground. Adhesive was placed on the object locations in the arena to secure objects in place. Between each trial, the arena and objects were disinfected with 70% ethanol. The trials were approximately 30 mins apart for each mouse. The four trials were habituation [in an empty arena, during which open field (OF) data was collected], training 1 (with two identical objects), training 2 (with the same identical objects), and test (with one of the objects replaced with a novel object). Objects were no taller than 50 mm, and were counterbalanced with each group. Objects were wooden and painted high-contrast colors, such as napkin rings and miniature candlesticks. Data for open field was quantified as the distance traveled (activity), and percent of time spent in the inner four squares of the arena grid (anxiety-related behavior, reviewed in Prut and Belzung, 2003). Data for the NOR was quantified as percent of time spent in each third of the arena and was verified for accuracy using video recordings. NOR was quantified using a discrimination index (DI): in which time spent with the novel object is “N” and time spent with the old object is “O,” a mouse’s discrimination index is N − O/(N + O). Mice were tested on all measures the day after tibial fracture surgery and were retested 2 weeks after surgery.

### Bromodeoxyuridine Injection

All mice were given two bromodeoxyuridine (BrdU; ThermoFisher Scientific, Waltham, MA, United States) injections: 1 and 10 h after surgery. Each injection contained 200 mg/kg of BrdU in 0.9% saline (Balu et al., 2009).

### Immunohistochemistry

Mice were sacrificed at either 1 day or 2 weeks after tibial fracture surgery. Under anesthesia, the thoracic cavity was opened, and the right atrium of the heart was cut. A solution of ice-cold 0.1 M phosphate-buffered saline (PBS) was perfused into the left ventricle of the heart. The brains were harvested and fixed in 4% paraformaldehyde for 3 days, then cryoprotected in 30% sucrose for 3 more days. Then, brains were freeze-mounted in OCT (VWR International, Radnor, PA, United States), and sliced on a cryostat at −20°C in 40 μm sections in a 1:4 series. Free-floating sections were suspended in PBS with 0.1% sodium azide preservative.

Briefly, sections that included the dorsal, not ventral, dentate gyrus (DG) of the hippocampus (bregma −1.28 through −2.12; Rosen et al., 2000; 4–5 sections identified using the visual resemblance of hippocampal shape to the Rosen et al., 2000 mouse brain atlas) were washed with PBS before quenching with a solution of 50% methanol (VWR) and 3% hydrogen peroxide (VWR) for 30 mins. Sections were washed again in PBS and blocked in a solution of 5 mL 0.01 M PBS, 150 μL normal donkey serum (Jackson ImmunoResearch, West Grove, PA, United States) and 50 μL TX100 (Sigma-Aldrich, St. Louis, MO, United States) at 20°C for 1 h and rinsed again in PBS. Sections were incubated at room temperature in one of three combinations of primary antibodies: to analyze microglia, a 1:500 concentration of goat anti-Iba1 primary antibody (Novus Biologicals, Littleton, CO, United States); to analyze astrocytes, a 1:500 concentration of rabbit anti-GFAP (glial fibrillary acidic protein; Santa Cruz Biotechnology, Inc., Dallas, TX, United States); to analyze neurons and cell differentiation/survival, a 1:200 concentration of rabbit anti-doublecortin (DCX) antibody (Cell Signaling Technologies, Danvers, MA, United States) and a 1:500 concentration of goat anti-BrdU primary antibody (Abcam, Cambridge, United Kingdom). The next day, sections were washed in PBS and incubated for 2 h in secondary antibody of donkey-anti-rabbit AlexaFluor 647 (ThermoFisher) and/or donkey-anti-goat AlexaFluor 488 (ThermoFisher) at a 1:200 concentration. Sections were washed in PBS before they were mounted onto slides, coverslipped using a fluorescent medium (Vectashield; Vector Labs, Burlingame, CA, United States), and sealed with nail polish. Z-stack images were collected on a Leica SP8 microscope (Leica Camera, Wetzlar, Germany) in the Duke University Light Microscopy Core Facility with 40× objectives. Images were taken as 150 μm × 150 μm “tiles,” which were then merged together to view the entire hippocampus (Supplementary Figure 1). Cells were quantified using manual cell counting (Iba1, hilar DCX, BrdU), unbiased stereology (DCX in SGZ) or densitometry (GFAP) using ImageJ software (NIH, Bethesda, MD, United States).

### Unbiased Stereology

Unbiased stereology for DCX+ cells in the subgranular zone of the dorsal dentate gyrus was conducted based on a previous protocol using ImageJ (NIH; Ip et al., 2017). A 150 μm × 150 μm counting frame was utilized in five sections that were sliced in a series of four. Optical fractionator estimates were multiplied by 2 to account for both hemispheres. Contours were traced around the granule cell layer, excluding the hilus.

### Densitometry

Astrocytic density was examined using densitometry as previously described for microglial density (Bilbo and Tsang, 2010). Densitometry is a measure of antigen expression, but this measure cannot be used alone to differentiate between an increase in astrocyte number and an increase in cell size. Using ImageJ software, signal pixels were defined as pixels within the dentate gyrus with a gray value 3 standard deviations higher than the mean gray value of a cell-poor area within the dentate gyrus (for region of interest, see Supplementary Figure 1B). The number of signal pixels and their average gray values above background were multiplied together to give an integrated density measurement for each section. Two to four sections were assessed per animal, with each section analyzed in horizontal sections every 2 microns.

### Statistical Analysis

Data were analyzed using two-way ANOVA and Tukey HSD *post hoc* test, when appropriate, with JMP Pro version 12.2 (SAS Institute, Cary, NC, United States). A two-way ANOVA between treatment (control diet + naïve, control diet + tibial fracture, or choline-supplemented diet + tibial fracture) and time point (1 day or 2 weeks post-surgery) was utilized in all measures. All graphs were generated using Prism version 8.3.0 (GraphPad, San Diego, CA, United States) using the grouped table format. Normality was confirmed for each outcome measure using the Anderson-Darling test in Prism.

## Results

### Dietary Choline Prevents Hippocampal-Dependent Postoperative Cognitive Decline

First, we evaluated the effects of 3 weeks of choline supplementation on hippocampal-dependent memory function using the NOR paradigm. Memory was assessed 1 day after surgery, and 2 weeks after fracture. No differences in total time exploring objects were found; all mice spent between 159 and 289 s in total exploration time during the test trial. Additionally, an aggressive ROUT analysis (*Q* = 10%) was conducted to ensure that there were no outliers skewing the means. We used the discrimination index [DI: (Novel object time − Old object time)/(Novel object time + Old object time)] to assess whether mice spent more time with the novel *versus* the old object (Ennaceur and Delacour, 1988). A significant effect of treatment on DI was observed at both time-points (*F*_2,37_ = 5.57, *p* < 0.01, Figure 3B). Following tibial fracture surgery mice could not recall the familiar object and interacted with both familiar and novel objects more equally. In contrast, mice consuming a supplemented choline diet for 3 weeks prior to tibial fracture had no difficulty recalling the familiar object and their memory was equal to the control mice. In the same cohort of mice, we also evaluated general activity and found that neither tibial fracture nor dietary choline supplementation altered mice locomotion (*F*_2,41_ = 2.48, *p* = 0.10, Figure 3C). This finding suggests that potential motor deficits caused by tibial fracture in the mice did not impair their ability to investigate objects. Interestingly, during the open field test, mice with tibial fractures spent less time in the center of the arena (*F*_2,41_ = 10.95, *p* < 0.001, Figure 3D), indicating an increased level of anxiety (Prut and Belzung, 2003). This increase in anxiety-related behavior was significant 1 day after surgery and was apparent (though not significant) 2 weeks after surgery. In contrast to its effects on memory, dietary choline supplementation did not mitigate this decrease in time spent in the center of the open field. Taken together, these results suggest that dietary choline prevents tibial fracture surgery-induced impairments in this hippocampal-dependent object recognition memory task.

### Tibial Fracture Surgery Impacts Young Neurons and Triggers Aberrant Neuronal Migration

**FIGURE 4:**
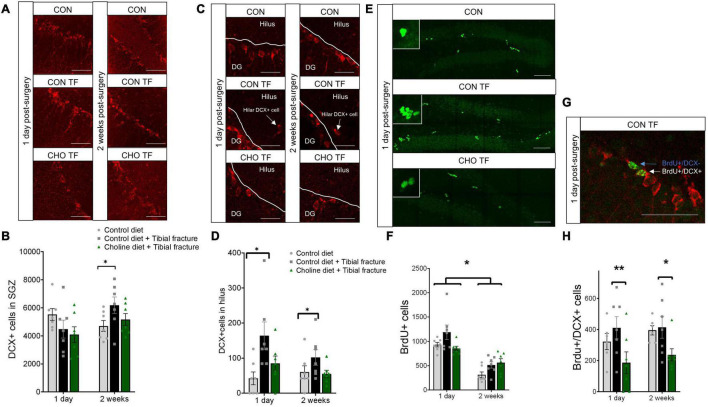
**(A)** Representative fluorescent micrographs of the SGZ taken 1 day and 2 weeks after surgery, immunostained with doublecortin (DCX), presented as maximum intensity projections of the Z-stack. CON, control diet + naïve; CON TF, control diet + tibial fracture surgery; CHO TF, choline diet + tibial fracture surgery. Scale bar: 50 μm. **(B)** Histogram showing the number of DCX+ cells in the subgranular zone (SGZ) of the dentate gyrus. *N* = 7 per group. *p < 0.05, Tukey HSD. Each bar represents the mean ± SEM. **(C)** Representative fluorescent micrographs of the SGZ taken 1 day and 2 weeks after surgery, immunostained with DCX, presented as a single image plane in the Z-stack. Scale bar: 25 μm. CON, control diet + naïve; CON TF, control diet + tibial fracture surgery; CHO TF, choline diet + tibial fracture surgery. **(D)** Histogram showing the number of DCX+ cells in the hilus of the dentate gyrus. *N* = 7 per group. *p < 0.05, Tukey HSD. Each bar represents the mean ± SEM. **(E)** Representative fluorescent micrographs of BrdU+ cells in the SGZ, taken 1 day after surgery. Two-week images not shown. Scale bar: 100 μm. **(F)** Histogram showing the number of BrdU+ cells in the SGZ of the dentate gyrus. *N* = 7 per group. *p < 0.05, Tukey HSD. Each bar represents the mean ± SEM. **(G)** Representative fluorescent micrograph of a BrdU+/DCX+ cell (white arrow) and a BrdU+/DCX− cell (blue arrow). Scale bar: 50 μm. **(H)** Histogram showing the number of BrdU+/DCX+ cells in the SGZ of the dentate gyrus. *N* = 6–7 per group. *p < 0.05, **p < 0.01, Tukey HSD. Each bar represents the mean ± SEM.

Young neurons in the subgranular zone (SGZ) of the dentate gyrus (DG) were identified using immunohistochemical staining for DCX after sacrifice either 1 day or 2 weeks after tibial fracture. There was a significant treatment × time point interaction (*F*_2,36_ = 3.34, *p* < 0.05, Figures 4A,B). No changes in numbers of DCX+ neurons were seen 1 day after surgery. In contrast, tibial fracture led to a 31.5% upregulation in DCX+ cells 2 weeks after surgery.

Aberrant neuronal migration was measured by counting DCX+ cells in the hilus of the hippocampus (ectopic granule cells). After combining both time points, there was a significant increase in hilar granule cells (*F*_2,36_ = 7.09, *p* < 0.01, Figures 4C,D) in the control diet + surgery group compared to the control diet + naïve group. No significant effect of dietary choline was found.

We also examined the fate of cells born within 10 h of tibial fracture surgery by administering BrdU 1 and 10 h post-surgery. While no significant effect of condition was observed at either timepoint (*F*_2,36_ = 0.17, *p* > 0.05, Figures 4E,F), there were significantly fewer BrdU+ cells 2 weeks after labeling (*F*_1,36_ = 62.54, *p* < 0.0001). There was also a marginal interaction between treatment and time point (*F*_2,36_ = 3.21, *p* = 0.05), because the decrease in BrdU+ cell number was lower in the choline-supplemented group (control group: 66.4% decrease; control diet + tibial fracture: 56.8% decrease; choline diet + tibial fracture: 34.0% decrease).

To further investigate the fate of the dividing neurons, a double-label of BrdU and DCX was exhaustively quantified. A significant difference due to treatment, but not time point, was found in dividing neurons (*F*_2,34_ = 6.41, *p* < 0.01, Figures 4G,H). Choline supplementation decreased the number of BrdU+/DCX+ cells. No differences due to time point (*F*_1,34_ = 0.76, *p* > 0.05) nor an interaction between treatment and time point (*F*_2,34_ = 0.18, *p* > 0.05) were observed. Double-labeled cells were also quantified in the hilus of the dentate gyrus, but an extreme paucity of double-labeled cells were observed (data not shown).

### Postoperative Astrogliosis Is Reduced in Mice Treated With Dietary Choline

**FIGURE 5:**
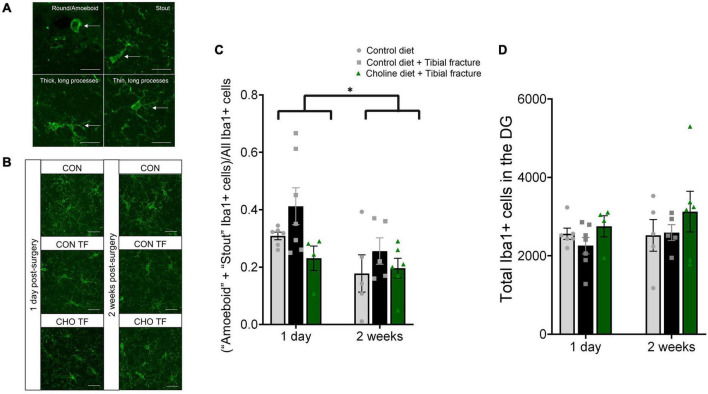
**(A)** Representative images of each microglial morphology type quantified. Sections were immunostained with Iba1. Scale bar: 10 μm. White arrows indicate each morphology type: round/amoeboid (*upper left*); stout (*upper right*); thick, long processes (*bottom left*); and thin, long processes (*bottom right*). Quantification states based on Schwarz et al. (2012). **(B)** Representative images of microglial morphology, represented as maximum intensity projections of the full Z-stack. Scale bar: 25 μm. **(C)** Histogram showing the percentage of “round” + “stout” Iba1+ cells in the dentate gyrus of the hippocampus 1 day and 2 weeks after surgery. *N* = 4–7 per group. *p < 0.05. **(D)** Tibial fracture and choline diet did not affect the total number of microglia. Histogram showing the total number of Iba1+ cells in the dentate gyrus 1 day and 2 weeks after surgery. *N* = 4–7 per group. Each bar represents the mean ± SEM.

To assess the activation state of neuroimmune cells, microglial activation in the DG was analyzed using immunohistochemical staining for Iba1. Activation was quantified as the percent of microglia in “activated” stages (“amoeboid” and “stout”) as previously described (Schwarz et al., 2012; Figures 5A,B). There was a significant effect of time point on microglial activation in the dentate gyrus (*F*_1,27_ = 6.86, *p* < 0.05, Figure 5C). One day after tibial fracture surgery, a higher percentage of microglia were in an “activated” state compared to 2 weeks after tibial fracture. This is consistent with previous findings indicating microglial activation resolves 1 week after tibial fracture (Cibelli et al., 2010). There was also a marginal effect of treatment (*F*_2,27_ = 3.22, *p* = 0.06, Figure 5C). Critically, differences were only found in the percent of microglia in “activated” morphologies, not total microglial number, in the dentate gyrus (*F*_2,27_ = 0.25, *p* > 0.05, Figure 5D). *Post hoc* analyses indicated that choline supplementation showed a trend (*p* = 0.06, Tukey’s multiple comparisons test) in reducing the number of round and amoeboid microglia in the DG of mice with tibial fractures, while there was no difference between the choline supplemented and naïve group at either timepoint.

**FIGURE 6:**
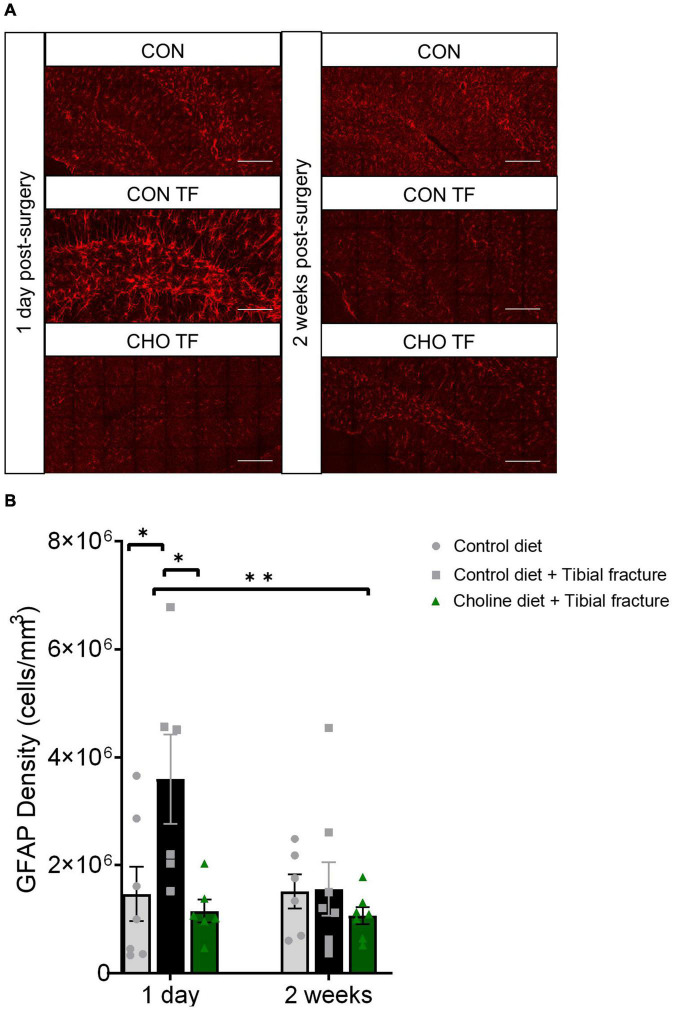
Tibial fracture and choline diet effects on astrocytic density. **(A)** Representative fluorescent micrographs of the dentate gyrus taken 1 day and 2 weeks after surgery, immunostained with glial fibrillary acidic protein (GFAP). Images are maximum intensity projections of the full Z-stack. CON, control diet + naïve; CON TF, control diet + tibial fracture surgery; CHO TF, choline diet + tibial fracture surgery. Scale bar: 100 μm. **(B)** Histogram showing the antigen density of GFAP in the dentate gyrus of the hippocampus. *p < 0.05, ^**^p < 0.01, Tukey HSD. Each bar represents the mean ± SEM. *N* = 6–8 per group.

To further assess neuroinflammation, astrocytic antigen density was analyzed using immunohistochemical staining and densitometry for GFAP after sacrifice either 1 day or 2 weeks after tibial fracture. A significant effect of treatment was observed in GFAP antigen density (*F*_2,34_ = 5.27, *p* < 0.05, Figure 6). Tibial fracture led to a significant increase in GFAP density, which was rescued with the addition of dietary choline supplementation. One day after tibial fracture, dietary choline supplementation led to the complete amelioration of increases in astrocytic density. As well, 2 weeks after surgery, no effects of fracture or choline were seen. This finding corroborates previous research showing that 1 day following tibial fracture, GFAP expression is increased in the hippocampus (Xu et al., 2017).

## Discussion

The aim of this study was to determine whether dietary choline supplementation could mitigate memory, neuronal, and neuroinflammatory deficits due to peripheral surgery. Our first goal was to quantify differences in memory 2 weeks after tibial fracture surgery, and test whether dietary choline impacts longer-lasting memory impairment. The second goal was to quantify young neurons in the hippocampus as a function of surgery, dietary choline supplementation, and time. Finally, we determined if the anti-inflammatory actions of dietary choline supplementation can mitigate the short-term inflammation at 1 day post-tibial fracture, when microglial activation is at its peak (Cibelli et al., 2010). We found that choline supplementation was effective in curtailing signs of postoperative neurocognitive disorder-like behavior and astrocytic density.

Astonishingly, 2 weeks after tibial fracture, hippocampal-dependent memory was still impaired, even though there was no longer any evidence of neuroinflammation. Dietary choline supplementation prior to and following tibial fracture surgery prevented the memory impairments that occurred both 1 day and 2 weeks following tibial fracture. Additionally, the largest cellular effect seen as a result of tibial fracture and dietary choline supplementation was in astrocytic density. Although hippocampal-dependent memory impairment was apparent at both time points examined, cellular measures differed based on the time point assessed. Specifically, increases in astrocytic density were seen 1 day after tibial fracture, but increases in young neurons in the SGZ were seen 2 weeks after tibial fracture surgery. Some alterations were persistent through both time points, such as increased numbers of hilar neurons. The largest neuroimmune effect of astrocytic density was found 1 day after tibial fracture, supporting the view that PND-associated neuroinflammation is transient. This research supports a dichotomous mechanism, in which immediate neuroinflammation is responsible for short-term impairments and some other mechanism – which may be delayed neuronal maturation, or altered connectivity due to hilar granule cells – may be responsible for the longer-term cognitive impairment seen in these studies.

A critical behavioral finding in the current work points to specific hippocampal actions of dietary choline supplementation: tibial fracture led to decrease in percent time spent in the center of an open field, and dietary choline supplementation did not prevent this decrease. Therefore, while choline supplementation mitigated the deficits in hippocampal-dependent memory caused by the tibial fracture, this treatment did not prevent the tibial fracture-induced anxiety-like behavior. Dietary choline supplementation appears to specifically rescue memory, and this knowledge can be used to further examine mechanisms underlying memory restoration. Because anxiety begins with increased excitation in the central amygdala (Ahrens et al., 2018), these findings suggest that choline is acting *via* a hippocampal, not amygdalar, mechanism. However, analysis of other regions, such as the amygdala, is needed to confirm this hypothesis in this model.

The current research showed significant effects of a non-seizure stimulus on hilar granule cells. Status epilepticus leads to a dramatic increase in neurogenesis, and some of these new neurons migrate not along the granule cell layer but instead enter the hilus (Parent et al., 1997; reviewed in Scharfman et al., 2007). Previous work has shown that status epilepticus leads to persistent impairments in hippocampal-dependent memory (Wong-Goodrich et al., 2010). Aberrant neuronal migration could be the cause of these deficits (Scharfman et al., 2000; Cho et al., 2015), lending support for the hypothesis that ectopic granule cells and the resulting aberrant neural circuitry may underlie the long-term cognitive deficits seen following tibial fracture. However, the role of ectopic granule cells in this model is not yet known. Additionally, to our knowledge, this is one of the first findings showing that a peripheral surgery can lead to this phenomenon, and the number of hilar granule cells in the present model is smaller than that seen after seizure. Of critical importance, no noticeable seizures were observed in the current study. We do not propose that tibial fracture surgery induces seizures, and hence ectopic granule cells. The present findings suggest that, even in the absence of seizures, peripheral assaults may increase the number of ectopic granule cells.

Though previous work has analyzed hippocampal DCX in the tibial fracture model of PNDs (Zhang et al., 2016), the current work assesses it at a more distant time point, and is the first to find increases in young neurons due to peripheral surgery. Though the current work found only a trending decrease in young neurons 1 day after tibial fracture, previous work has shown a decrease in DCX+ cells in the SGZ 1 day after tibial fracture (Zhang et al., 2016). However, 2 weeks after surgery, more young neurons were observed in the SGZ after tibial fracture. Critically, there was not an increase in BrdU+/DCX+ cells due to surgery alone, indicating that the increase in DCX+ may not be an increase in neurogenesis, but a delay in neuronal maturation. This result suggests that tibial fracture leads to delayed neuronal maturation. DCX is a marker for *young* neurons. These neurons, under non-pathological conditions, mature into “fully-grown” neurons that express other markers, like NeuN (Shepherd et al., 2016). An increase in DCX+ may mean there is a developmental block; the same rate of neurogenesis is occurring, but because fewer of these cells are maturing, there is a “buildup” of these young cells. This interpretation is also supported by the lack of surgery-induced increases in BrdU+/DCX+ cells 2 weeks after surgery, indicating that these young neurons did not newly divide after surgery. This explanation is further supported by the decrease in BrdU+/DCX+ cells in the choline-supplemented group. Cells dividing at the time of surgery in the choline treatment group presumably continued to differentiate into mature neurons, which would not be labeled with DCX. The majority of the BrdU+ cells that are not also DCX+ are also not IBA+ or GFAP+ (data not shown), indicating that they did not differentiate into glial cells and are likely to be mature neurons.

A delayed maturation phenotype is consistent with other models of neural assault. Previous work a model of electroconvulsive seizure has shown enhanced or delayed neuronal development, depending on the timing of the insult (Ueno et al., 2019). Conditions that increase likelihood of seizures, such as focal cortical dysplasia (Lee and Kim, 2013) and aging (Kotloski et al., 2019), have shown similar decreases in neuronal maturation (Rao et al., 2005; Hanai et al., 2010). Further study analyzing the lifespan of these neurons, using markers for neuronal stem cells and mature neurons, is needed to confirm whether this is a blunting of maturation. However, this possible delay in neuronal maturation matched the seizure-like phenotype shown in hilar granule cells. Unlike other significant effects reported here, an increase in young neurons was observed only 2 weeks after tibial fracture surgery. For this reason, it is a possible mechanism for long-term PNDs: an increase in young neurons promotes too much forgetting (Akers et al., 2014) and impedes hippocampal-dependent memory. However, further research is needed to determine the functional role of these cells.

Additionally, adult dietary choline supplementation significantly prevented the increase in GFAP antigen density. This is consistent with previous work indicating that the central “cholinergic anti-inflammatory pathway” also includes astrocytes (Gamage et al., 2020). However, the increase in astrocytic density due to surgery does not elucidate the source of increased GFAP reactivity: an increase in astrocyte division (Robel et al., 2011), and/or an increase in cell size (Sun and Jakobs, 2011) may contribute to this increase in GFAP. However, the increase in GFAP implicates one or both of these sources of increased astrocytic inflammation. Previous work has implicated astrocytes in PNDs (Xu et al., 2017). Specifically, the chemokine CCL2, expressed by astrocytes, causes microglial activation and cognitive dysfunction following tibial fracture surgery (Xu et al., 2017). This specific chemokine has been shown to be downregulated by cholinergic agonists in peripheral tissues (Wang et al., 2011; Li et al., 2018). Whether the increase in astrocytic density results from earlier neuroimmune events and circumstances (Clarke et al., 2018) remains unknown in this model.

Although the increase in astrocytic density due to surgery was reduced by dietary choline supplementation 1 day after surgery, the polarization state of these astrocytes is unclear. Though choline supplementation may be dampening the activation state or the number of astrocytes, it is also possible that dietary choline supplementation dampens astrocyte recruitment without affecting astrocytic activation. Alternatively, choline could be altering astrocyte polarization state with no affect on astrocyte recruitment. Further analysis into the specific actions of dietary choline supplementation on astrocyte polarization is required.

Microglial activation, as quantified my morphology, was not significantly increased due to surgery. Importantly, physical morphology indicates a difference from the “ramified” state; however, morphological state cannot differentiate between M1 (“pro-inflammatory”) and M2 (“anti-inflammatory”) microglia, which excrete different cytokines and serve different functions (reviewed in Subhramanyam et al., 2019). Microglia are heterogenous, and show different transcriptional profiles across brain regions as a function of age (Grabert et al., 2016) and sex (Villa et al., 2018). The current work does not differentiate between the different types of microglia that show a non-ramified morphology, and these “amoeboid” and “stout” microglia may be M1, M2, or some combination of these. Future studies are required to assess the differences between transcriptional states due to tibial fracture surgery and dietary choline supplementation.

Previous work has associated postoperative memory deficits with infiltrating macrophages from the periphery due to the breakdown of the blood-brain barrier (Terrando et al., 2011; Degos et al., 2013; Vacas et al., 2014). This phenomenon is ameliorated after microglial ablation, indicating that the process of macrophage infiltration due to injury relies on microglia, the resident macrophages of the brain (Feng et al., 2017). However, the current work conflicts with previous findings, as no significant microglial effects were observed due to surgery, but memory deficits persisted.

Choline supplementation only significantly affects DI and astrocytic density. Though a choline-supplemented diet does not completely prevent the effects of tibial fracture on DCX+ cells in the SGZ and hilus, the results are not statistically different from either the naïve control or the control diet + tibial fracture group, possibly indicating partial protection. Small sample size may be one of the reasons for the lack of significance. Additionally, the lack of significant differences indicate that choline supplementation has subtle effects. Further work using the bolus dose of choline should be conducted to see if the present findings can be replicated, and to examine if a bolus dose would lead to dramatic neuronal effects.

The mechanism by which dietary choline exerts the effects described here is currently unknown. Dietary choline may affect brain and behavior *via* multiple actions. First, ACh levels in the brain may increase after dietary choline supplementation, directly impacting neurogenesis and other neural changes (Cohen and Wurtman, 1976; Klein et al., 1991). Second, dietary choline may offset the drop in systemic choline after surgery (Ulus et al., 1998). Third, dietary choline supplementation may be acting *via* increases in the number of anatomically localized receptors and agonists that are difficult to detect systemically or *via* whole-brain assessment (Morley et al., 1977; Coutcher et al., 1992; Guseva et al., 2012). Future work is required to pinpoint the specific mechanistic action, or combination of actions, of dietary choline supplementation. Additionally, though this work focuses on the DG, it does not focus on other subregions of the hippocampus, which may exhibit different inflammatory reactions to tibial fracture and dietary choline supplementation; further work is needed to confirm that these effects are specific to the DG and not to other subregions.

One additional possible explanation for these subtle differences is the role of the blood-brain barrier (BBB), which is likely of importance in these findings. Previous work has shown that tibial fracture leads to a breakdown of the BBB (Terrando et al., 2011). As well, dietary choline supplementation alters choline transport across the BBB (Wecker and Trommer, 1984). How these two models interact to affect the BBB is currently unknown.

One remaining question involves risk and resilience. PNDs affect a subset of people, and the current data suggests that dietary choline may protect against it. Individual differences in susceptibility to PNDs may be tied to underlying differences in the cholinergic system and its interaction with the neuroimmune system. Due to dietary choline’s critical role in neurodevelopment and previous work showing long term cognitive “programming effects” (Meck and Williams, 1999, 2003; Wu et al., 2012; Blusztajn and Mellott, 2013), and the shown interaction between choline supplementation and the neuroimmune system in development (Wu et al., 2015), early-life choline could be protective against later-life PNDs. The potential protective effects of supplementation prior to surgery, or even throughout the lifespan, may also impact likelihood and prevalence of PNDs.

In summary, these findings provide interesting new evidence that there may be multiple neuro-immune mechanisms underlying the short- and longer-term cognitive impairment seen after tibial fracture surgery. These data also provide encouraging evidence that consuming a choline-rich diet both before and after peripheral surgery may be a sufficient treatment to combat PNDs.

## Data Availability Statement

The raw data supporting the conclusions of this article will be made available by the authors, without undue reservation.

## Ethics Statement

The animal study was reviewed and approved by Duke University Institutional Animal Care and Use Committee.

## Author Contributions

SM, NT, and CW conceived and designed the experiments and wrote the manuscript. SM and CK performed the experiments. All authors contributed to the article and approved the submitted version.

## Conflict of Interest

The authors declare that the research was conducted in the absence of any commercial or financial relationships that could be construed as a potential conflict of interest.

## Publisher’s Note

All claims expressed in this article are solely those of the authors and do not necessarily represent those of their affiliated organizations, or those of the publisher, the editors and the reviewers. Any product that may be evaluated in this article, or claim that may be made by its manufacturer, is not guaranteed or endorsed by the publisher.

## Supplementary Material

Supplementary Figure 1 “Stitched” images of “tiles” indicating the counting area in the dentate gyrus of the hippocampus, presented as maximum intensity projections of the Z-stack. **(A)** Fluorescent micrograph of the dentate gyrus showing DCX+ staining. White box indicates an example of cells that would be quantified. **(B)** Fluorescent micrograph of the dentate gyrus showing GFAP staining. White outline indicates region of interest.
