# Supplementary figures and images for: Dietary Choline Protects Against Cognitive Decline After Surgery in Mice
Source: Front Cell Neurosci. 2021 Dec 14;15:671506. doi: 10.3389/fncel.2021.671506 (PMC8712952; doi:10.3389/fncel.2021.671506)

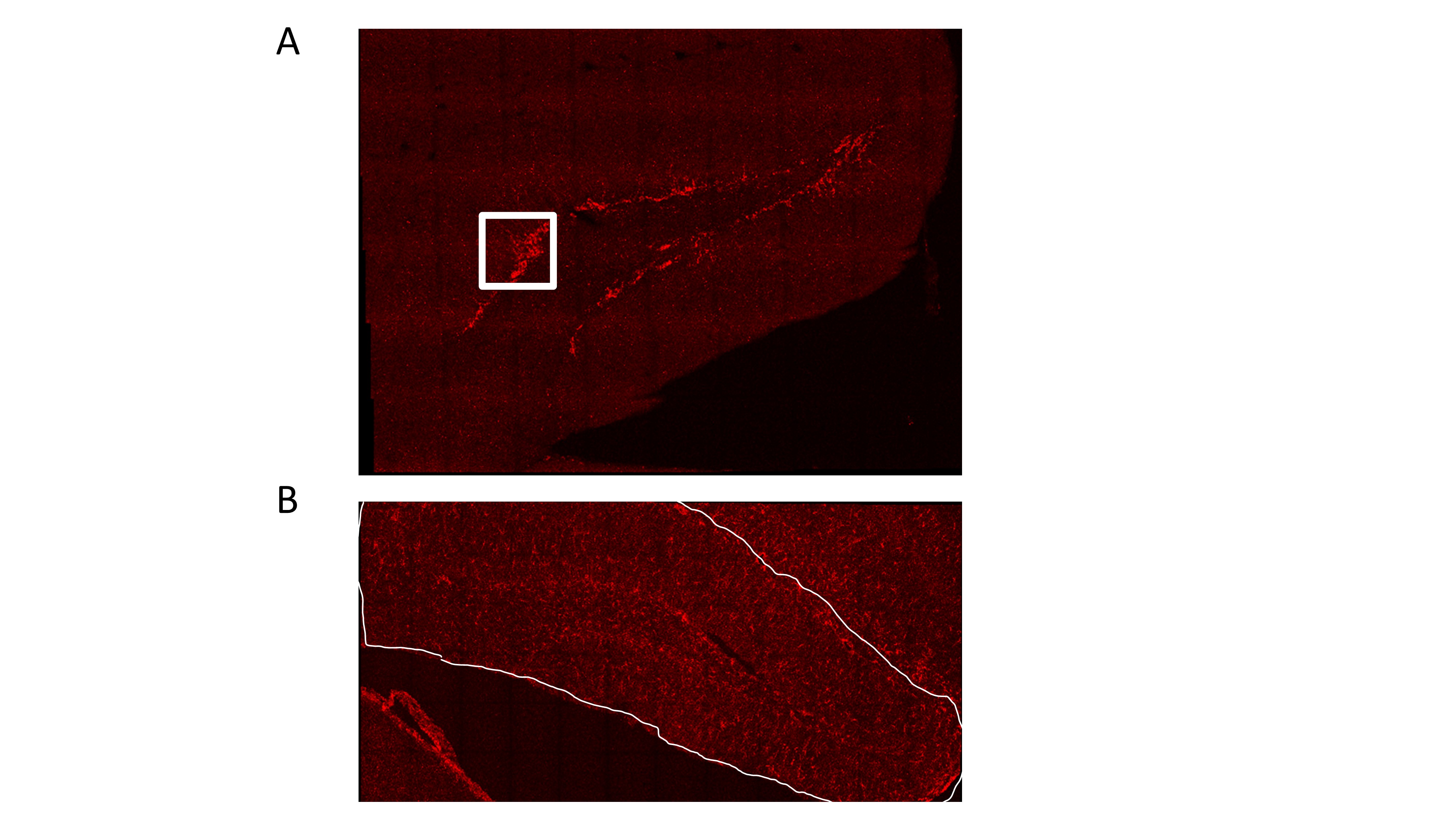

Supplement: Supplementary Figure 1 — “Stitched” images of “tiles” indicating the counting area in the dentate gyrus of the hippocampus, presented as maximum intensity projections of the Z-stack. (A) Fluorescent micrograph of the dentate gyrus showing DCX+ staining. White box indicates an example of cells that would be quantified. (B) Fluorescent micrograph of the dentate gyrus showing GFAP staining. White outline indicates region of interest. [file Image_1.jpg]
